# Calcium‐mediated DAD in membrane potentials and triggered activity in atrial myocytes of ETV1^f^ ^/^ ^f^MyHC^Cre^ ^/+^ mice

**DOI:** 10.1111/jcmm.70005

**Published:** 2024-08-19

**Authors:** Li‐Hua Fang, Qian Chen, Xian‐Lu Cheng, Xiao‐Qian Li, Tian Zou, Jian‐Quan Chen, Guo‐Jian Xiang, Qiao Xue, Yang Li, Jian‐Cheng Zhang

**Affiliations:** ^1^ Shengli Clinical Medicine College of Fujian Medical University Fuzhou Fujian China; ^2^ Department of Critical Care Medicine Division Four Fujian Provincial Hospital Fuzhou Fujian People's Republic of China; ^3^ Department of Cardiology Nanping First Hospital Affiliated to Fujian Medical University Nanping Fujian People's Republic of China; ^4^ Department of Cardiology Fujian Provincial Hospital Fuzhou Fujian People's Republic of China; ^5^ Department of Cardiology, the Sixth Medical Center Chinese People's Liberation Army Hospital Beijing People's Republic of China

**Keywords:** atrial fibrillation, calcium spark, DADs, ETV1, RyR2, SCaE

## Abstract

The E‐twenty‐six variant 1 (ETV1)‐dependent transcriptome plays an important role in atrial electrical and structural remodelling and the occurrence of atrial fibrillation (AF), but the underlying mechanism of ETV1 in AF is unclear. In this study, cardiomyocyte‐specific ETV1 knockout (ETV1^f/f^MyHC^Cre/+^, ETV1‐CKO) mice were constructed to observe the susceptibility to AF and the underlying mechanism in AF associated with ETV1‐CKO mice. AF susceptibility was examined by intraesophageal burst pacing, induction of AF was increased obviously in ETV1‐CKO mice than WT mice. Electrophysiology experiments indicated shortened APD_50_ and APD_90_, increased incidence of DADs, decreased density of *I*_Ca,L_ in ETV1‐CKO mice. There was no difference in V_INACT,1/2_ and V_ACT,1/2_, but a significantly longer duration of the recovery time after inactivation in the ETV1‐CKO mice. The recording of intracellular Ca^2+^ showed that there was significantly increased in the frequency of calcium spark, Ca^2+^ transient amplitude, and proportion of SCaEs in ETV1‐CKO mice. Reduction of Cav1.2 rather than NCX1 and SERCA2a, increase RyR2, p‐RyR2 and CaMKII was reflected in ETV1‐CKO group. This study demonstrates that the increase in calcium spark and SCaEs corresponding to Ca^2+^ transient amplitude may trigger DAD in membrane potential in ETV1‐CKO mice, thereby increasing the risk of AF.

## 1 INTRODUCTION

Atrial fibrillation (AF) is the most common tachyarrhythmia. As of 2016, AF has affected nearly 46.3 million people worldwide.^1^ AF significantly increases the incidence of thromboembolic events, heart failure and sudden cardiac death.^2^, ^3^ Large cohort studies have shown that genetic factors account for 40%–62% of the susceptibility risk for AF.^4^ Recent studies showed that the E‐twenty‐six variant 1 (ETV1)‐dependent transcriptome played an important role in atrial electrical and structural remodelling and the occurrence of AF.^5^, ^6^ Rommel et al.^7^ found that atrial ETV1 mRNA and protein expression were higher in patients with the AF compared to the sinus rhythm. cardiomyocyte‐specific knockout ETV1 mice have abnormal cardiac function and electrophysiology, along with P wave extension, His‐Purkinje system development delay, reduced gap junction conduction, decreased sodium channel current density and altered gating mechanism.^8^ However, the atria of cardiomyocyte‐specific overexpressing ETV1 mice showed large amounts of interstitial fibrosis, increased collagen fibrils, disordered sarcomere, decreased myocardial cell mitochondria, raised marker gene‐actin of vimentin and fibroblasts, and occurrence of irregular RR interval, and P wave loss, enhanced atrial arrhythmias and mortality, while ventricular size and function showed normal.^7^ Thus, Fatkin^9^ regarded the ETV1 as a new participant in atrial remodelling and the occurrence of AF. The above results strongly suggest that the transcription factor ETV1 plays an important role in the maintenance of atrial structure and conduction system, and is closely related to the occurrence of AF.

At present, the theory of multiple wavelets, local trigger and rotor are recognized widely, and can partially explain the pathogenesis of AF.^10^ But the mechanism of AF remains unclear in detail. The occurrence of AF must have two factors: the one is ectopic trigger focus which include early after depolarization (EAD), delayed after depolarization (DAD) and 4 phase depolarization^11^; the other is abnormal myocardial substrate, abnormal substrate refers to altered electrical conduction between cardiomyocytes.^11^, ^12^, ^13^, ^14^, ^15^ Calcium ions play a crucial role in the excitation‐contraction coupling of the heart. To contract the atria, a storage compartment within heart cells known as the sarcoplasmic reticulum releases calcium ions into the main compartment of the cells. Calcium ions also enter the cell from the surrounding tissue. As the atria relax, calcium ions are pumped back into the sarcoplasmic reticulum or out of the cell by specific transport proteins.^16^ Intracellular Ca^2+^ abnormalities play an important role in the formation of ectopic rhythms, conduction abnormalities and AF.^17^, ^18^, ^19^ Abnormal release, reuptake and overload of calcium ions and reduction of L‐type calcium channel current (*I*_Ca,L_) in atrial myocytes are the cellular electrophysiological basis of AF.^20^, ^21^ It is clear that abnormal intracellular calcium regulation plays a crucial role in AF.^22^ So far, however, whether ETV1 can regulate intracellular calcium remains unclear.

Rommel et al.^7^ showed that the ETV gene is necessary for angiotensin II (Ang II) which induced atrial remodelling. ETV1 is initiated by G‐protein coupled receptors and activated by the RAS‐MAPK (Mitogen Activated Protein Kinase) pathway; ETV1 can also serve as a transcription target downstream of Ang II induced MAPKs activation, participating in atrial remodelling. Cardiomyocyte‐specific deficiency of ETV1 could block the effect of Ang II, but not mentioned the protein of calcium regulation‐related proteins.^5^, ^23^ Based on the above results, we hypothesized that ETV1 may directly or indirectly affect calcium regulation, cause increased intracellular calcium load, increase ectopic rhythm and induce AF. Therefore, in this study, cardiomyocyte‐specific ETV1 knockout (ETV1‐CKO) mice were constructed to observe the susceptibility to AF and the underlying mechanism in AF associated with ETV1‐CKO mice.

## 2 MATERIALS AND METHODS

### 2.1 Animal model generation

The cardiomyocyte‐specific ETV1 null mice, hereby referred to as ETV1‐CKO mice (ETV1^f/f^MyHC^Cre/+^), were generated using CRISPR/Cas9 technique by Saiye Biotechnology Co., LTD (Suzhou, China). Mice were housed and cared at PLA General Hospital Laboratory (Beijing, China). All our experimental procedures were approved by the Ethics Committee of Fujian Provincial Hospital and performed in accordance with the Guide for the Care and Use of Laboratory Animals published by the US National Institutes of Health (publication no. 23, revised 1996).

### 2.2 Electrophysiology study

Mice were anaesthetized using 1% sodium pentobarbital (average dose, 30 mg pentobarbital per kg of body weight) injected into the left peritoneal cavity 5 min before electrical recording. Intraperitoneal injection of sodium pentobarbital was used as a method of euthanasia (Average dose, 5.4 g pentobarbital per kg of body weight). A subcutaneous II‐lead ECG of was used to obtain ECG recordings in the in vivo studies. Intraesophageal burst pacing was used to assess the susceptibility to AF. AF was defined as a rapid, fragmented f‐waves with irregular AV node conduction and ventricular rhythm lasting for at least 10 s immediately after a burst pacing cycle. The stimulation voltage was two times the diastolic threshold, the pulse width was 2 ms. First paced for 10 s with a circle length of 100 ms, followed by burst stimulation for 10–12 s with a circle length of 30 ms, until 10 consecutive burst stimulation or AF was induced. The occurrence and duration of AF in each group were observed and recorded.

### 2.3 Haematoxylin and eosin staining and Masson staining

Atrial structure and fibrosis changes were observed by haematoxylin and eosin staining and Masson staining.

Atrial muscle cells were immersed in 4% paraformaldehyde for 4 h and then transferred to 70% ethanol. Individual lobes of atrial myocyte biopsy material were placed into a treatment cassette, dehydrated through a series of alcohol gradients, and embedded in paraffin blocks. Prior to immunostaining, 4 μm tissue sections were dewaxed in xylene and washed in PBS by reduced ethanol rehydration followed by haematoxylin and eosin staining. After staining, sections were dehydrated by increasing concentrations of ethanol and xylene.

In the Masson staining, the sections were dewaxed to water and fixed in Bouin solution overnight, and rinsed with running water until the sections were colourless. It was counterstained with Weigert's haematoxylin for 10 min, fully washed and turned blue. Place the spring red liquid for 5 min and rinse slightly in distilled water. 1% phosphomolybdate acid was coloured for 5 min and the microscopic control cytosol and myofibers appeared in bright red. Bright green dye solution was then stained for 3,5,7 and 10 min. 1% acetic acid 1 min, 95% alcohol and 100% alcohol were dehydrated.

### 2.4 Echocardiography

Mice were weighed and anaesthetised with isoflurane inhalation (3% for induction, 1% for maintenance). The anterior chest was shaved, and the mice were placed on a heating pad in the left lateral decubitus position. A rectal temperature probe was placed to ensure that the body temperature remained at 37.0°C during the study. Left ventricle structure and function were assessed by previously validated two‐dimensional M mode and Doppler echocardiographic techniques. Echocardiographic images were obtained using a Visual sonic VEVO 2100 ultrasound system. Digital images were analysed offline according to modified American Society for Echocardiography standards. LV end‐diastolic and end‐systolic diameters and anterior and posterior wall thickness in diastole were measured from M‐mode tracings obtained at the midpapillary level. LV ejection fraction was derived from M‐mode parameters. LV mass was estimated from the M‐mode data, and LV end‐diastolic and end‐systolic volumes were calculated using the formula of Teichholz et al.^24^ Analysis of data was performed by an investigator blinded to the treatment assigned.

### 2.5 Single atrial myocyte preparation

Mice were anaesthetised by CO_2_/O_2_ and sacrificed by cervical dislocation, after which the heart was excised, cannulated, mounted on a Langendorff set‐up, and perfused at 37°C for 4–5 min with Ca^2+^‐free Tyrode's solution containing (in mmol/L): 140 NaCl, 4 KCl, 1 MgCl_2_, 5.5 glucose, 10 HEPES, with the pH adjusted to 7.40 with NaOH. Then, the isolated heart was perfused with Tyrode's solution containing 1 mg/mL collagenase II (Worthington, USA) and 0.25 mg/mL trypsin (Gibco, USA) for 11–13 min. The left and right atrial appendages were removed and digested in the enzyme solution to form single atrial cells. Then, isolated atrial myocytes were stored in a low Ca^2+^ (0.5 mM) Tyrode's solution. Finally, atrial myocytes were moved to a Tyrode's solution containing 1.8 mM Ca^2+^ for measuring intracellular Ca^2+^ and membrane currents/potentials.

### 2.6 Confocal Ca^2+^ imaging and intracellular Ca^2+^ quantification

Confocal calcium imaging was used to detect intracellular Ca^2+^ changes by recording Ca^2+^ sparks and Ca^2+^ waves as well as the timing of Ca^2+^ peak and Ca^2+^ decay. Fluo‐4 AM (Thermo Fisher Scientific, USA), a non‐ratio metric Ca^2+^ indicator that is commonly used with 488 nm excitation, was added to acute isolated atrial myocytes and incubated in black light avoidance boxes. After de‐esterification, a laser‐scanning confocal microscope (SP5, Leica Microsystems, German) was used to record spontaneous Ca^2+^ release events (SCaEs) and the constant rate of Ca^2+^ cycling under different conditions. Small dishes were placed in the confocal microscope fixation slot and given S1S1 stimulation with 1 Hz, and Ca^2+^ sparks and Ca^2+^ waves were observed. Then using Myocyte Calcium & Contractility Recording System (IonOptix, USA), S1S1 stimulation with 1 Hz was given to observe Ca^2+^ release and Ca^2+^ relevant indicators: Ca^2+^ release amplitude (△F/F0), half time for Ca^2+^ release (*t* to peak 50%), half time for Ca^2+^ elimination (*t* to bl 50%), 90% time for Ca^2+^ release (*t* to peak 90%), 90% time for Ca^2+^ elimination (*t* to bl 90%), elimination time constant (sin exp tau), etc. Among them, △F/F0 and *t* to peak 50% reflect Ca^2+^ release function of sarcoplasmic reticulum, *t* to bl 50% and sin exp tau reflect the sarcoplasmic reticulum Ca^2+^ recovery function.

### 2.7 Patch clamp experiments

Patch‐clamp experiments were used to record action potentials and L‐type *I*_Ca,L_ in atrial myocytes. The Axon Multiclamp 700B Amplifier (Molecular Devices, USA) was connected to the computer. Signal acquisition was completed using a digidata 1440A acquisition interface (Molecular Devices, USA) controlled by pCLAMP programs (version 10.2).

To record the action potential, cells were kept at 37°C and bathed in a solution that contained NaCl 140 mM, CaCl_2_ 1.8 mM, MgCl_2_ 1 mM, HEPES 10 mM, KCL 4 mM and glucose 5.5 mM; it was at pH 7.40 and was adjusted with NaOH. The glass pipettes were filled with a solution of KCl 20 mM, kaspartate 120 mM, MgCl_2_ 1 mM, HEPES 10 mM, Na_2_ATP 4 mM and glucose 10 mM at pH 7.3, which was adjusted with KOH.

*I*_Ca,L_ were recorded at 37°C. Tetrodotoxin (5 μM) was added to block sodium current when recording calcium currents. The pipette solution contained CsCl_2_ 120 mM, CaCl_2_ 2 mM, MgCl_2_ 5 mM, EGTA 11 mM, HEPES 10 mM, and Na_2_ATP 5 mM, adjusted to pH 7.2 with CsOH. The external solution contained NaCl 140 mM, CaCl_2_ 2 mM, MgCl_2_ 1 mM, KCl 4 mM, HEPES 10, and glucose 10 mM, adjusted to pH 7.4 with CsOH.

Action potentials were recorded after giving a 2.5 ms/1 nA depolarization stimulation under current‐clamp mode. Calculate the resting membrane potential (RMP), AP duration at 50 and 90% of repolarization (APD_50_ and APD_90_, respectively) and triggered activity. Triggered activity was defined as an unstimulated action potential arising from a DAD or an EAD. The *I*_Ca,L_ was obtained under the voltage clamp mode. *I*_Ca,L_ was recorded from a holding potential of −40 mV to +50 mV with a 10 mV voltage step, and the depolarization pulse was set to 200 ms. Current amplitude data of each cell were normalized to its cell capacitance (current density, pA/pF) and current voltage relationship (I–V curve) was plotted. Voltage‐dependent activation and steady‐state inactivation profiles were fitted to a Boltzmann equation *a* = 1/{1 + exp[−(Vm−V½)/*k*]}, where *a* is the normalized conductance, V_m_ is the test potential, V½ is the potential at which current is half activated/inactivated, and *k* is the slope factor. Electrophysiological data were analysed by Clampfit 10.4 (Axon Instruments) and Origin (Microcal software).

### 2.8 Western blot analysis

Western blot was used to investigate the expression of the ion channel proteins Cav1.2, NCX1.1, and the calcium transport‐related proteins RyR2, p‐RyR2 (Ser2808, Ser2814), SERCA2a and CaMKII. Total protein was obtained from 12‐week‐old ETV1‐CKO mice and WT mice. Protein samples were equally loaded on 8% sodium dodecyl sulphate (SDS) polyacrylamide gels and transferred onto nitrocellulose membranes. The membranes were blocked with 5% non‐fat milk for 1 h at room temperature and then incubated with the specified primary antibody overnight at 4°C. Protein blots were performed under primary antibody incubation including anti‐RyR2 (abcam), anti‐p‐RyR2 (abcam), anti‐CaMKII (CST), anti‐ETV1 (Invitrogen), anti‐NCX1.1 (abcam) and anti‐SERCA2a (abcam). After 1‐h incubation with the required secondary antibody, the particular signals were revealed by the chemiluminescence detection reagent Western lightning plus‐ECL. Western blotting data densitometric analysis was conducted using Image J software.

### 2.9 Statistical analysis

Data were processed using Origin 8.5 and Image J software. The data were presented as the mean ± SD, with *n* denoting the number of cells analysed. One‐way ANOVA with Bonferroni post hoc analysis or student's *t*‐test was used for comparison between groups. Fisher's exact test was used to compare the incidences of atrial tachyarrhythmia and oscillation in membrane potentials (DADs/EADs) between the groups. SPSS 17.0 was used for analyses, with *p* < 0.05 considered statistically significant.

## 3 RESULTS

### 3.1 Myocyte‐specific knockout of ETV1 expression

**FIGURE 1:**
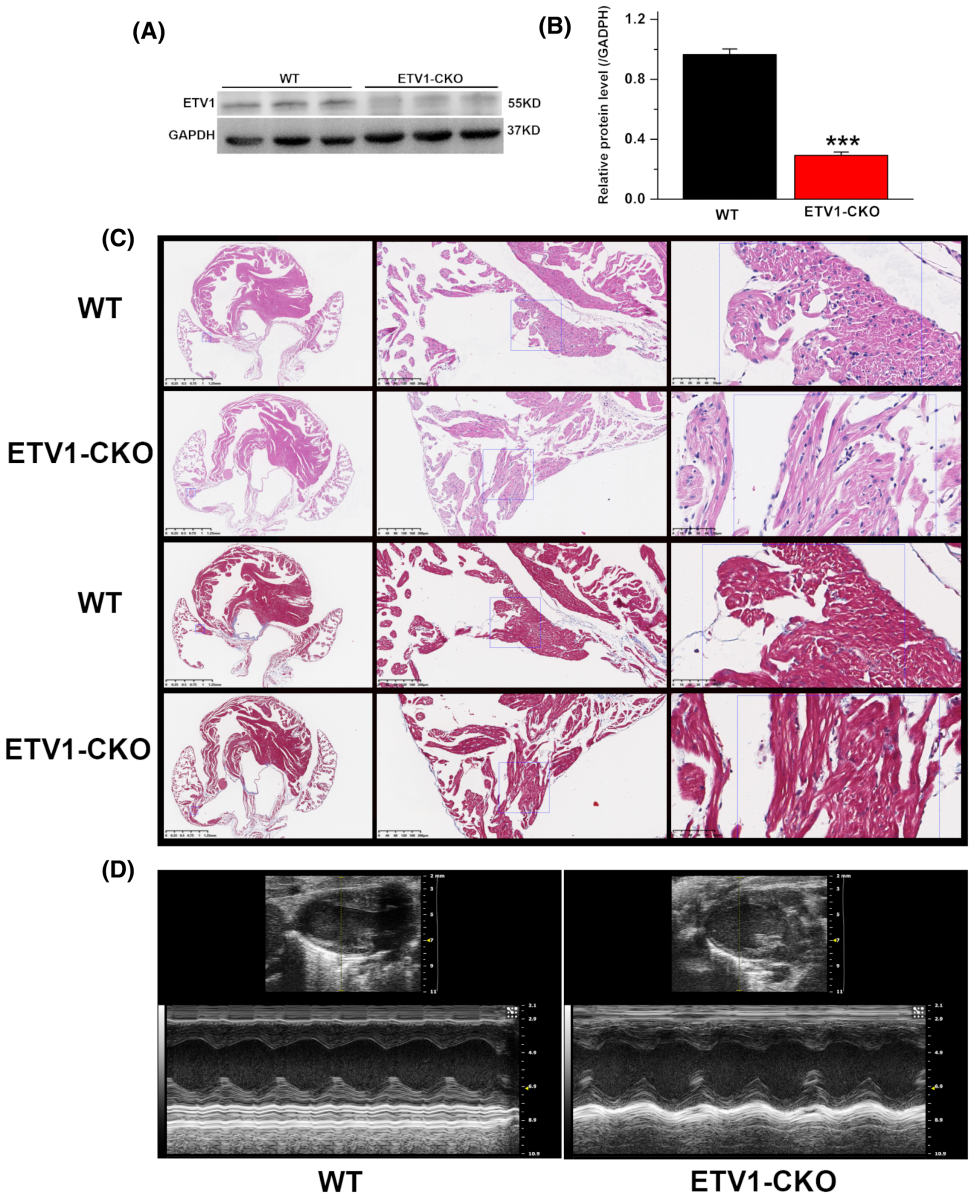
Cardiomyocyte morphology of specific ETV1‐CKO mice. (A, B) Protein level of ETV1 in the atria of the two groups mice. Molecular weight on western blot: ETV1 ~55 kDa (values are presented as mean ± SD. *n* = 3, ***p < 0.001 vs. WT, by student's *t*‐test). (C) Heart tissue of two groups. Haematoxylin and eosin staining showed no significant morphological differences in cardiac four lumen sections between ETV1‐CKO and WT mice. Masson staining showed no evidence of atrial fibrosis (*n* = 3 per group at 16–20 weeks old, scale bars of the left is 1.25 mm, the middle is 200 μm, the right row of rulers is 50 μm). (D) Representative M‐mode images showing systolic excursion of LV walls in ETV1‐CKO mice (left), indicating reduced systolic function compared with their WT littermates (right).

ETV1 floxed C57BL/6J mice were crossed with transgenic MyHC‐Cre C57BL/6J mice to produce cardiomyocyte‐specific ETV1‐knockout mice (ETV1^f/f^MyHC^Cre/+^, ETV1‐CKO). Atrial tissue immunoblotting showed that ETV1‐CKO mice reduced ETV1 protein levels compared with WT mice (Figure 1A,B). The results showed that compared with WT, the level of ETV1 in ETV1‐CKO mice decreased by 73%.

Haematoxylin and eosin staining showed that the muscle fibres in the atrial myocardium of the two groups were arranged neatly, and there was no obvious muscle fibre rupture or inflammatory cell infiltration. Masson staining showed no obvious atrial fibrosis in the two groups (Figure 1C).

Representative M‐mode tracings from echocardiography are shown in Figure 1D. ETV1‐CKO mice did exhibit slight declines in LV function, as determined by reductions by 2% in ejection fraction (*p* > 0.05).

### 3.2 Increases the susceptibility to AF in ETV1‐CKO mice

**FIGURE 2:**
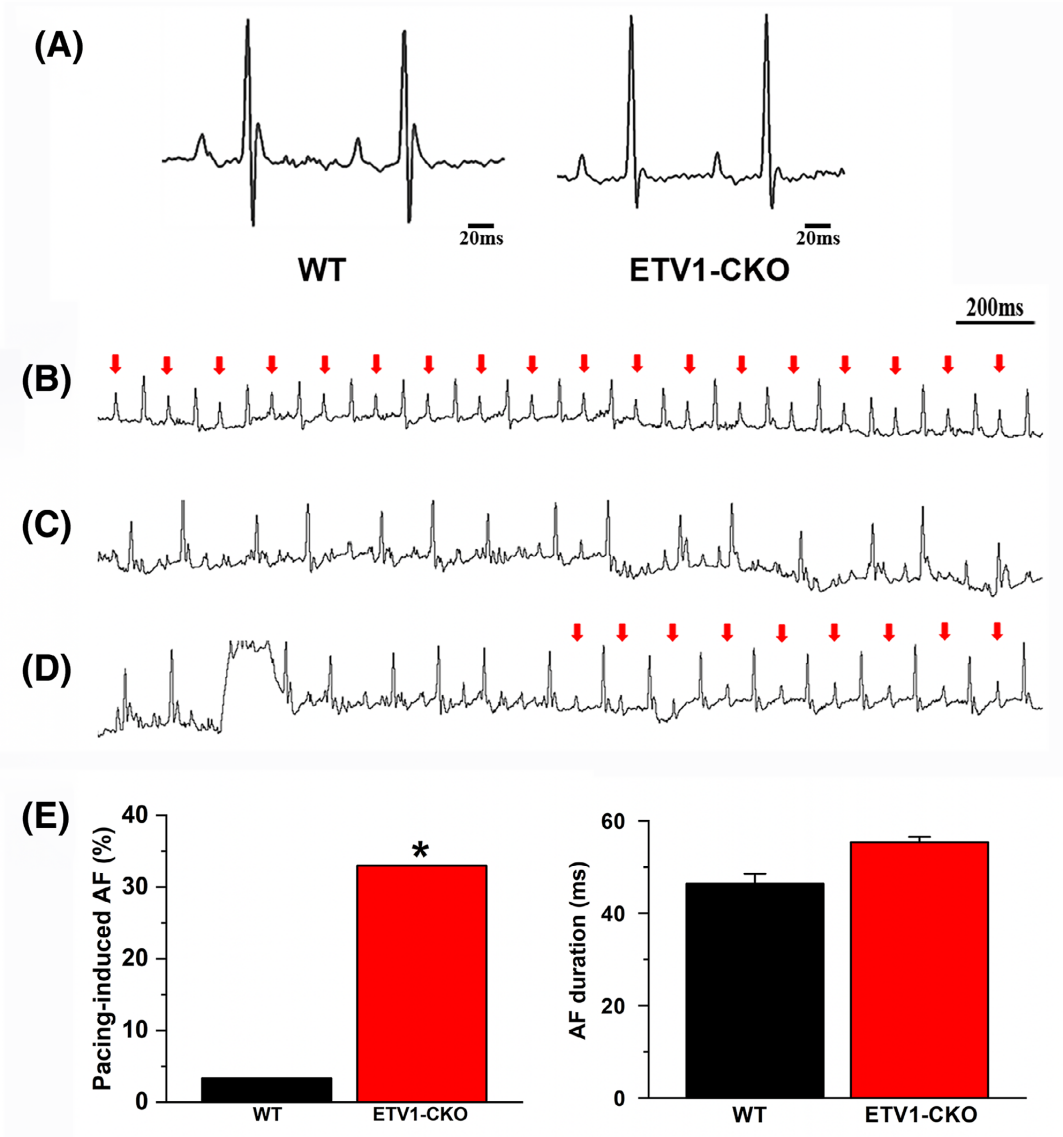
ECG before and after rapid inducing in ETV1‐CKO mice. (A) Representative recordings of surface ECG lead II showing no change in baseline ECG parameters comparing 4‐month‐old ETV1‐CKO and WT mice. (B) Red arrows showed sinus rhythm. (C) Induced AF. (D) Spontaneously restore sinus rhythm from AF. (E) Bar graph summarizing the incidence of inducible AF in ETV1‐CKO mice. Data were analysed using Fisher's exact test (*p < 0.05 vs. WT). No difference was found in duration of AF between two groups (*n* = 40 mice for Etv1^f/f^MyHC^Cre/+^ and *n* = 30 mice for control).

Seventy‐two mice were used for transesophageal atrial pacing. Two mice were excluded from the analysis due to anaesthesia problems. Thirty WT mice and forty ETV1‐CKO mice were studied. Representative recordings of surface ECG lead II showed no change in baseline ECG parameters between 4‐month‐old ETV1‐CKO and WT mice (Figure 2A). The electrocardiograms before and after rapid inducing in ETV1‐CKO mice were shown in Figure 2B,D. AF and episodes of atrial flutter were labelled as AF (Figure 2C), AF was induced by 30 Hz pulse pacing in 12 of 40 (30%) ETV1‐CKO mice, just 1 of 30 (3.33%) in WT mice (*p* < 0.05) (Figure 2E). No difference was found in duration of AF between two groups (ETV1‐CKO 55.35 ± 1.18 ms vs. WT 46.42 ± 2.08 ms, *p* > 0.05).

### 3.3 Shortened APD and increased DAD in atrial myocytes of ETV‐CKO mice

**FIGURE 3:**
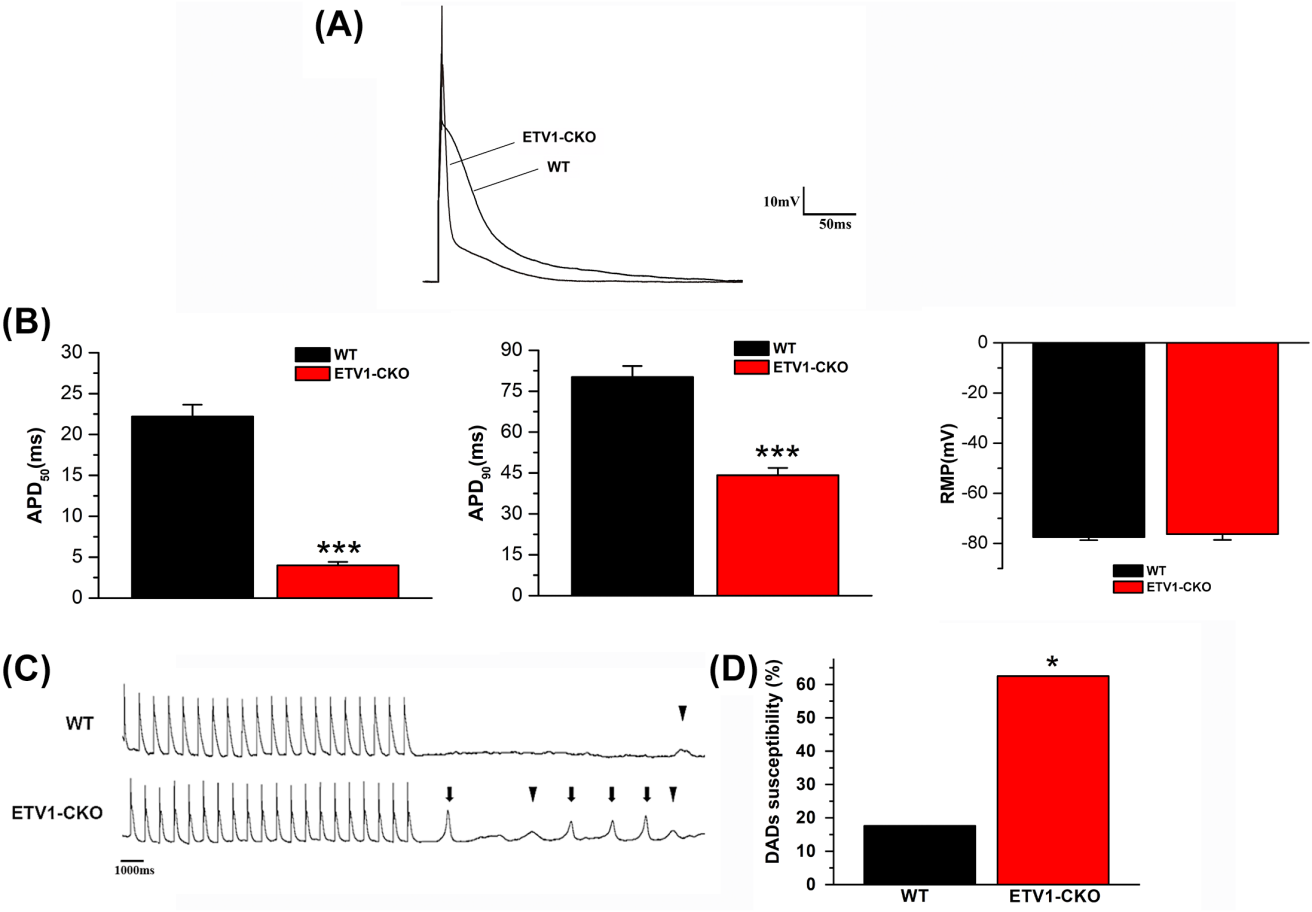
Action potential maps, DADs and TA plots of atrial myocytes in both groups. (A) Original plots of two groups of action potentials. (B) APD50 and APD90 were significantly shortened in the ETV1‐CKO group compared with the WT group (*n* = 16/3 cells/mice for Etv1^f/f^MyHC^Cre/+^ and *n* = 17/3 cells/mice for control, ***p < 0.001 vs. WT, by student's *t*‐test). No difference was found in RMP between two groups. (C) The action potentials DAD (▼) and spontaneous AP (**↓**) recorded in WT and ETV1‐CKO groups, respectively. (D) Increased atrial myocyte DADs in the ETV1‐CKO versus WT groups (*n* = 16/3 cells/mice for Etv1^f/f^MyHC^Cre/+^ and *n* = 17/3 cells/mice for control, *p < 0.05 vs. WT, by student's *t*‐test).

The action potentials of both groups were showed in Figure 3A. APD_50_ (ETV1‐CKO 4 ± 1.65 ms vs. WT 22.20 ± 5.38 ms, *p* < 0.001) and APD_90_ (ETV1‐CKO 44.18 ± 10.31 ms vs. WT 80.18 ± 15.79 ms, *p* < 0.001) were significantly shorter in the ETV1‐CKO group compared to WT (Figure 3B). No difference was found in RMP between two groups (ETV1‐CKO −77.50 ± 1.24 mV vs. WT −76.3 ± 2.29 mV, *p* > 0.05) (Figure 3B).

All electrical activities were recorded in 17 atrial cells from 5 ETV1‐CKO mice and 16 atrial cells from 5 WT mice at 1 Hz pacing (Figure 3C). The proportion of atrial cells in DADs was increased in the ETV1‐CKO group compared to the WT group (ETV1‐CKO 62.5% (10/16) vs. WT 17.6% (3/17), *p* < 0.05; Figure 3D).

### 3.4 The change of atrial myocytes *I*_Ca_ _,L_ in ETV1‐CKO and WT mice

**FIGURE 4:**
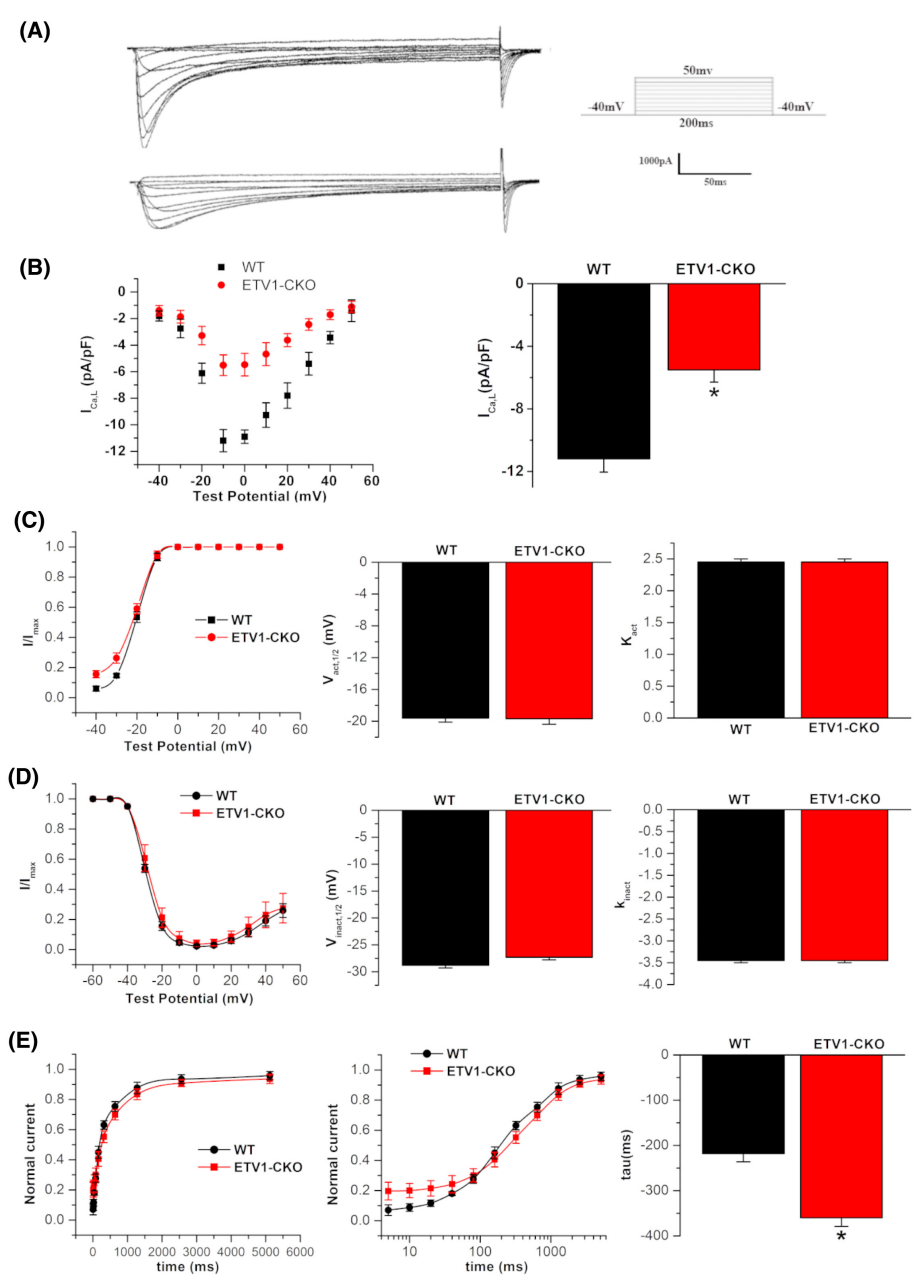
L‐calcium currents and the alteration of gating dynamics of *I*_Ca,L_ in atrial myocytes in both groups. (A) Raw diagram of L‐type calcium current in two groups. (B) I–V relationship curve of L‐type calcium currents recorded from Etv1^f/f^MyHC^Cre/+^ (*n* = 17/6 cells/mice) and control (*n* = 15/6 cells/mice) atrial cardiomyocytes. The amplitudes of L‐type calcium current were normalized to cell capacitance and presented as mean ± SD. I–V current curve showed “inverted bell type” in two groups. The bar plot illustrates comparison of peak of *I*_Ca,L_ density between two groups (*p < 0.05 vs. WT, by student's *t*‐test). (C, D) Steady‐state activation and inactivation curves, semi‐activation (half‐inactivation) voltage and curve slope (*n* = 17/6 cells/mice for Etv1^f/f^MyHC^Cre/+^ and *n* = 15/6 cells/mice for control). (E) Time course of recovery from inactivation, the tau value was significantly longer in ETV1‐CKO group as compared with the WT group (*n* = 16/6 cells/mice for Etv1^f/f^MyHC^Cre/+^ and *n* = 13/6 cells/mice for control) (*p < 0.05 vs. WT, by student's *t*‐test).

Current density in ETV1‐CKO was significantly reduced when compared to WT (Figure 4A). When the clamping voltage was set at 10 mV, the peak of the *I*_Ca,L_ showed a significant decrease in ETV1‐CKO compare to WT (ETV1‐CKO 5.50 ± 0.77 pA/pF vs. WT–11.19 ± 0.83 pA/pF, *p* < 0.05) (Figure 4B).

There was no significant difference in the voltage dependence of steady state activation and inactivation in L‐type calcium channel between the groups (Figure 4C,D), but recovered more slowly in ETV1‐CKO as compared with WT mice (ETV1‐CKO 359.99 ± 19.04 ms vs. WT 218.11 ± 18.19 ms) (Figure 4E).

### 3.5 Elevated incidence of calcium transients and spontaneous calcium release in atrial myocytes of ETV1‐CKO mice

**FIGURE 5:**
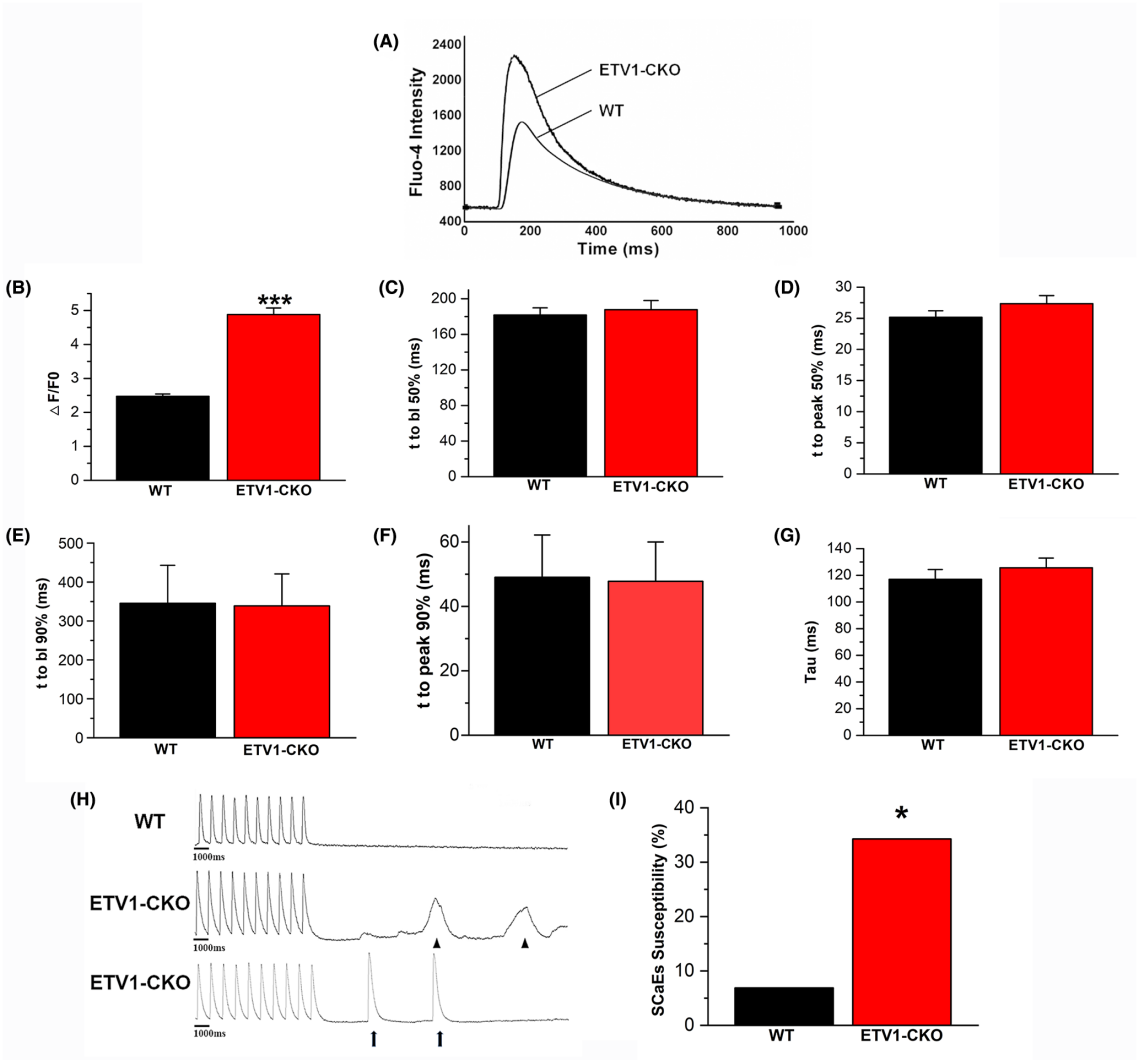
Calcium transients (CaTs) of atrial myocytes in the two groups. (A) Representative tracings of Ca^2+^ transient recordings during 1 Hz pacing. (B) The elevated amplitude of CaT in ETV1‐CKO group. (C–G) No difference in t to peak 50% (bl%), *t* to peak 90% (bl%) and calcium release decay time (peak time) between two groups. (H) DADs (▲) or spontaneous CaTs (**↑**); (I) Increased SCaE in the ETV1‐CKO group (34.28% (12/35) vs. 6.89% (2/29), *n* = 35/5 cells/mice for Etv1^f/f^MyHC^Cre/+^ and *n* = 29/5 cells/mice for control. *p < 0.05 vs. WT, ***p < 0.001 vs. WT, by student's *t*‐test).

Cardiomyocytes from WT and ETV1‐CKO mice underwent field stimulation at 1 Hz until the calcium transients (CaTs) reached a steady‐state. The pacing was then stopped for 10 s and CaTs were recorded in 35 atrial cells from seven ETV1‐CKO mice and 29 atrial cells from six WT mice, simultaneously, the incidence of SCaE was observed (Figure 5A). The results showed that the CaT amplitude increased significantly in the ETV1‐CKO group (Figure 5A,B), no significant differences in the time to baseline 50%, time to baseline 90%, time to peak 50%, time to peak 90% and calcium release decay time (tau) between the two groups (Figure 5C–G), and the proportion of SCaEs increased (34.286% (12/35) vs. 6.89% (2/29), *p* < 0.05) in the ETV1‐CKO group (Figure 5H,I).

### 3.6 Increased calcium spark in atrial cells of ETV1‐CKO group

**FIGURE 6:**
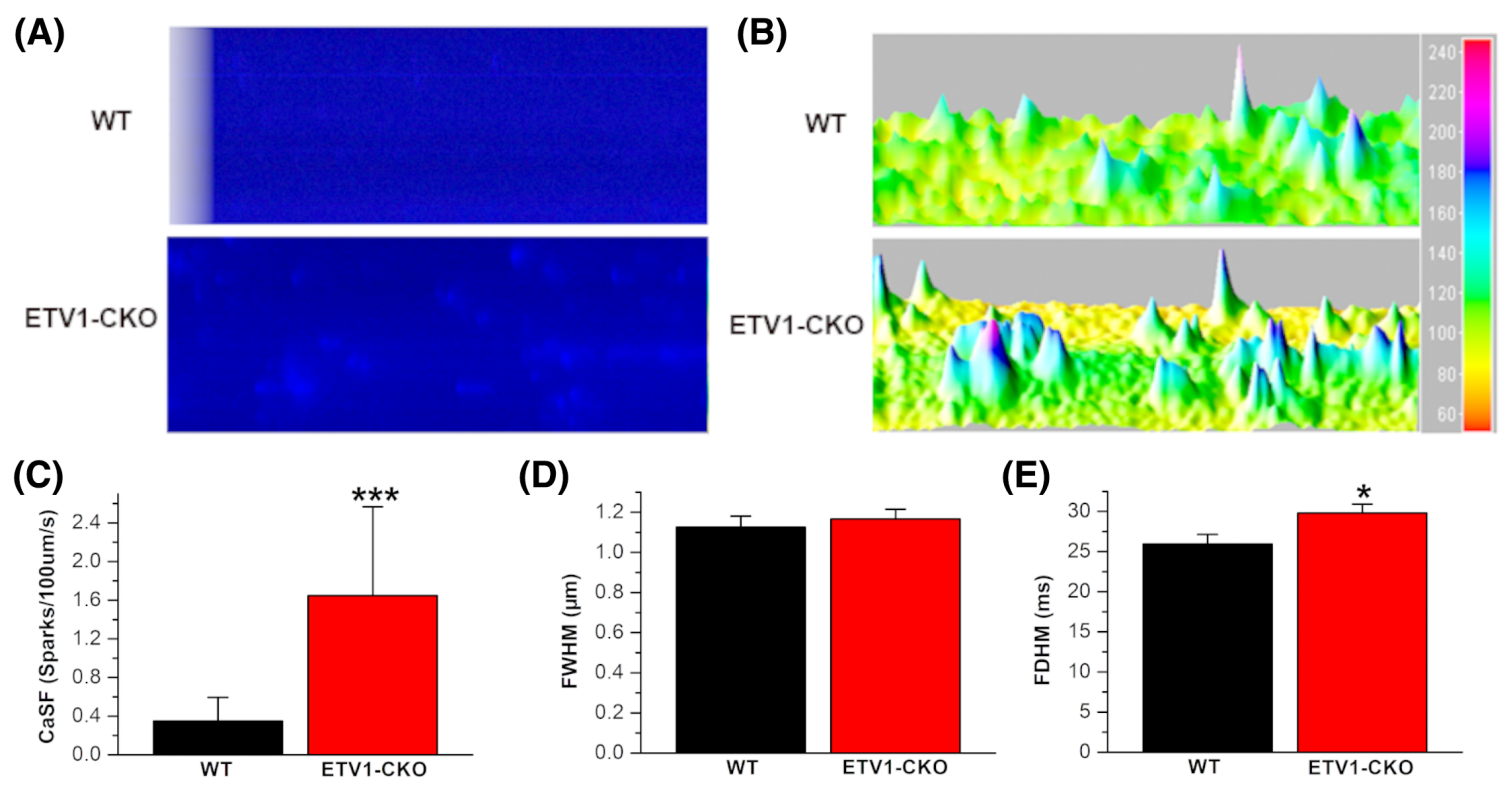
Calcium sparks of atrial myocytes in the two groups. (A) Calcium spark frequency in myocytes from WT and Etv1^f/f^MyHC^Cre/+^ mice pre‐conditioned by pacing at 1 Hz. (B) 3D imaging of calcium spark. (C) Calcium spark frequency between two groups (ETV1‐CKO 1.6467 ± 0.9196 sparks/100 μm/s vs. WT 0.348 ± 0.2449 sparks/100 μm/s, *n* = 40, *p* < 0.001). (D) FWHM: Half height width. (E) FDHM: Half height duration (*n* = 17/3 cells/mice for Etv1^f/f^MyHC^Cre/+^ and *n* = 15/3 cells/mice for control, ***p < 0.001 vs. WT, *p < 0.05 vs. WT, by student's *t*‐test).

To analyse the effect of ETV1‐CKO on calcium spark. Cardiomyocytes were paced at 1 Hz for 5 min until CaTs reached steady state, then pacing was stopped and diastolic calcium sparks were measured for 10 s in a 1.8 mM calcium solution. At 1 Hz pacing frequency, there was a higher frequency of calcium sparks in the ETV1‐CKO group as compared with the WT group (ETV1‐CKO 1.6467 ± 0.9196 sparks/100 μm/s vs. WT 0.3487 ± 0.2449 sparks/100 μm/s, *n* = 40, *p* < 0.001) (Figure 6A~C), Calcium Spark FDHM increased (29.79 ± 11.18 ms vs. 25.93 ± 9.19 ms, *n* = 40, *p* < 0.05). The calcium spark FWHM is almost unchanged (Figure 6D,E).

### 3.7 Expression of calcium channel and calcium transporter of atrial tissue in two groups

**FIGURE 7:**
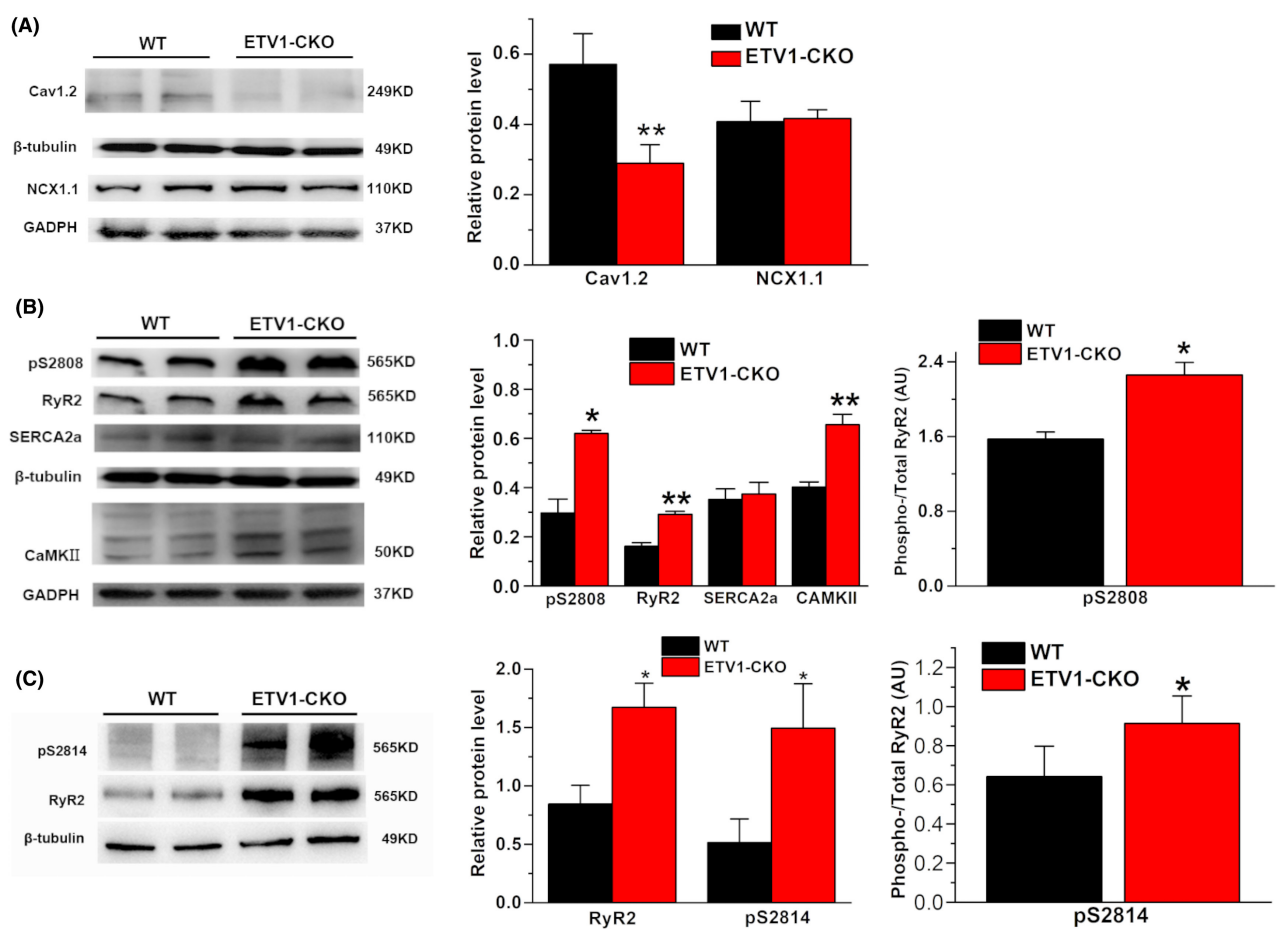
Calcium channel (A) and transporter protein (B,C) expressing in the atria of the two groups. Molecular weight on western blot: Cav1.2 ~249 kDa, NCX1.1 ~110 kDa, p‐RyR2 (S2808 and S2814) ~565 kDa, RyR2 ~565 kDa, SERCA2a ~110 kDa, CaMKII ~50 kDa, β‐tubulin ~49 kDa, and GAPDH ~37 kDa. (Values are presented as mean ± SD. *n* = 3, *p < 0.05 vs. WT, **p < 0.01 vs. WT, by student's *t*‐test).

The expression of Cav1.2 proteins, NCX1 (Figure 7A), calcium transport‐related proteins RyR2, p‐RyR2 (S2808 and S2814), SERCA2a and CaMKII (Figure 7B,C) were measured by western blot. The results showed that down‐regulated expression of Cav1.2, upregulated expression of RyR2, p‐RyR2, no change of expression of SERCA2a and NCX1 in ETV1‐CKO group compare with WT.

## 4 DISCUSSION

This study showed that there were increased susceptibility to AF, decreased *I*_Ca,L_, shortened APD, elevated the incidence of DADs, and increased the occurrence of SCaEs and calcium leakage in ETV1‐CKO mice. ETV1‐CKO causes a downregulation of Cav1.2 expression, which is the molecular basis of *I*_Ca,L_ reduction; upregulation of RyR2, p‐PyR2 and CaMKII expression is the molecular basis of increment in calcium release and calcium leakage. This study explored the relationship between ETV1‐CKO and AF, calcium disposal and the electrophysiological mechanisms of triggering activity.

### 4.1 Increased the susceptibility to AF in ETV1‐CKO mice

Intraesophageal burst pacing was used to assess the susceptibility to AF showed that there was increasingly incidence of susceptibility to AF by 30% in ETV1‐CKO mice, which was nine times that of WT mice. No significant difference was found in duration of AF maintenance time between two groups. A recent report by Yamaguchi N et al.^25^ found that a significant reduction expression of ETV1 in left atria in patients with lower LVEF and in mouse models of cardiac pressure overload, resulting in electrical remodelling and structural remodelling of the left cardiac chamber. This study focused on the role of ETV1 in atrial fibrosis and cardiac conduction related with the sodium current, while the relationship between ETV1 and AF is unclear. Our study deplores the possible mechanism of ETV1 deficiency increasing the susceptibility to AF from the perspective of calcium handling. Reversely, Rommel et al.^7^ reported that increased atrial ETV1 expression was found in human and mice. Overexpression of ETV1 led to atrial structural remodelling, which induced atrial arrhythmias; on contrary, cardiomyocyte‐specific knockdown of ETV1 expression can prevent Ang II mediated atrial remodelling. The study is quite opposite to those observed in this study, the reasons may be related to the following factors: (1) the few clinical samples in the study, only the right atrial appendage tissue from three cases of AF was used for RNA sequencing, protein expression analysis of the right atrial appendage tissue was analysed from nine cases of AF; (2) the sequence of exon 11 bp of mouse knockout, however, the ETV1 exon 14 bp gene sequence was knockout in our study, this may cause ETV1 protein function differently; (3) the tool mouse used in the ETV1‐deficient construction was MLC2a‐Cre C57BL/6J mouse in this study, however, the tool mouse used was αMyHC‐Cre C57BL/6J mouse in our study.

### 4.2 Shortened APD in ETV1‐deficient atrial myocytes, proning to re‐entry

The results of this study showed that APD_50_, APD_90_ were significantly shortened, underlying the chronic electrical remodelling of atrial myocytes, which is an important mechanism for the early maintenance of AF. The APD shortening mainly stems from the shortening of the action potential plateau phase, which mainly consists of the inward L‐type calcium current (*I*_Ca,L_) and outward potassium currents (*I*_Ks_, I_Kr_).^26^ Voltage clamp pattern recordings further revealed that *I*_Ca,L_ reduced in the atrial myocyte of the ETV1‐CKO group, while clamp voltage was 10 mV, the peak of *I*_Ca,L_ was reduced by 50%, so the APD shortening mainly resulted from the decreased *I*_Ca,L_ in atrial myocytes. Furthermore, the studies of the channel regulation mechanism revealed that both semi‐activated voltage V_act,1/2_ and a semi‐inactive voltage of V_inact,1/2_ remained unchanged in two groups, but the recovery time (tau) was significantly delayed in the ETV1‐CKO group, speculated that the recovery delay after inactivation was another one of the reasons for reduction of *I*_Ca,L_. To date, no related reports were found that ETV1 had impact on action potential and atrial myocyte *I*_Ca,L_.

### 4.3 The abnormal intracellular calcium disposition in ETV1‐deficient atrial myocytes, induced DADs

The results of this study demonstrated that DADs were significantly higher in ETV1‐CKO mice than WT mice, strongly suggested causal relationship between the susceptibility to AF and increased DADs; DADs are the most direct cellular electrophysiological basis for triggering the increase in AF.^27^ Haïssaguerre,^28^ in the early years, found that 90% of the ectopic trigger foci located in the left and right upper and inferior pulmonary veins in patients with paroxysmal AF. Our previous study on gene knock‐in mice (CASQ2R33Q/R33Q) of sarcoplasmic reticulum calsequestrin‐2 mutations also found an increased incidence of DADs and triggering activity.^22^ This is the origin of ectopic focus formation in AF. In the ETV1‐CKO group, we also observed that the recovery rate did not increase and the amplitude of DAD exceeded the threshold, and DAD produced trigger activity and depolarization in atrial myocyte. DAD formation results from an abnormal intracellular calcium disposition.

#### 4.3.1 Systolic CaT amplitude increased in the ETV1‐CKO group, RyR2 considered as a key molecular in AF

The CaT amplitude was significantly increased in the ETV1‐CKO group. The ETV1 deficiency affected the amplitude of CaTs but not the calcium time to peak or decay. The amplitude of CaTs indicates the amount of calcium released from sarcoplasmic reticulum to the cytoplasm. Calcium decay means the process of calcium recycling from the cytoplasm to sarcoplasmic reticulum. Increased CaT suggest increased calcium release from sarcoplasmic reticulum within a short peak time frame, while constant calcium decay suggest that the mechanisms for reuptake and exchange were proportionally enhanced to compensate for the elevated concentration of calcium ions within the same time frame, potentially causing intracellular calcium overload. The results of WB still showed that upregulation of RyR2 and increased number of p‐RyR2 (Ser 2808) in ETV1‐CKO group, no difference in the expression of SERCA and NCX1 between two groups. The increased amplitude of calcium release may be associated with the increased expression of RyR2. Increasing evidence suggests that the abnormal subcellular level of calcium signalling is a key factor in AF.^29^, ^30^ Intracellular calcium is not solely regulated by CICR, but is also correlated with expression, phosphorylation and dephosphorylation of the RyR2.^5^ RyR2 is a known susceptibility locus for AF, and RyR2 mutations are associated with AF.^31^, ^32^ Upregulation of RyR2 expression is more sensitive to calcium‐triggered calcium release, which explained the amplitude of calcium release more. Besides, upregulation of p‐RyR2 (Ser2808) identified as the physiological phosphorylation sites on RyR2 for PKA in ETV1‐CKO group, leading to dissociation of calstabin 2 (also known as FK506‐binding protein 12.6),^33^ increased RyR2 channel open probability and diastolic Ca^2+^ leak.

#### 4.3.2 Diastolic SCaE significantly increased in the ETV1‐CKO group, CaMKII‐dependent phosphorylation of RyR2 linked to AF

Calcium‐spark frequency was significantly higher in atrial myocyte from ETV1‐CKO mice than WT mice. Calcium spark FDHM increased, while calcium spark FWHM remained almost unchanged. Atrial myocyte Ca^2+^ sparks and free Ca^2+^ increased significantly and transient inward current (*I*_Ti_) also increased, led to the higher incidence of DAD and trigger activity, and eventually formed the ectopic rhythm origin in the atrium. The Voigt N Team^34^ studies showed that Ca^2+^‐I_NCX_ coupling gain was increased, as well as the higher sarcoplasmic reticulum of Ca^2+^ leak in the cardiomyocytes of patients with chronic AF, which increased the propensity for arrhythmogenic DADs/TA. It has been demonstrated that SCaEs burst more frequently in chronic AF, accompanied with membrane potential oscillations in the form of DADs and triggered action potentials.^35^ The size of the DAD depended on at least two factors: the latency and amplitude of the SCaE and the sensitivity of RMP (Vm) to the intracellular Ca^2+^.

In the disease state, the SCaE is observed when spontaneous calcium sparks or arrhythmogenic calcium waves are significantly enhanced.^36^, ^37^ This defective Ca^2+^ dynamic balance is usually caused by the remodelled calmodulin (CaM).^38^, ^39^ Clinically, the pathophysiological mechanism of spontaneous sarcoplasmic calcium release in patients involves the downregulation of CaM and its dysfunction. This study showed that CaM kinase (CaMKII) was increased in the atrial myocytes in ETV1‐deficient mice. CaM is a ubiquitous calcium‐binding protein. CaM regulates RyR2 directly by binding to RyR2 and indirectly via Ca^2+^‐CaM kinaseII (CaMKII)‐mediated phosphorylation of RyR2 at S2814. Phosphorylation of RyR2 by CaMKII enhances spark‐detected Ca^2+^ leak.^40^ Conversely direct binding of CaM to RyR2 has the opposite effect and reduces spark‐detected Ca^2+^ leak.^41^ The structural mechanisms of how CaM binding modulates RyR2 activity are not known, but it appears that RyR2 needs to be phosphorylated either at residue 2808 (PKA/CaMKII site) or S2814 (CaMKII site) for CaM to reduce spark frequency.^39^, ^42^ A study has reported that the RyR2 channel must be phosphorylated by CaMKII in order for CaM to exert an inhibitory effect, however, the same study implicitly indicates that CaMKII phosphorylation does not alter the mode of CaM binding or the affinity of CaM for RyR2. These results suggest the presence of a complex network of allosteric signalling used for transmitting structural changes imparted by CaM‐RyR2 binding to the RyR2 channel pore.^39^

CaMKII is a potential regulator of RyR2 function and can increase the open probability of RyR2 in the atrial myocytes at resting state, thereby increasing resting‐state calcium leakage or facilitating the occurrence of sarcoplasmic reticulum SCaEs.^19^, ^43^, ^44^ Ca^2+^ leakage was increased in sarcoplasmic reticulum at resting state in ETV deficiency mice, which may promote the occurrence of arrhythmogenic DADs. Therefore, ETV1‐CKO may lead to increased intracellular Ca^2+^ concentration in atrial myocytes by CaMKII and changing the Ca^2+^ permeability of RyR2 at the resting state, which is more likely to cause intracellular calcium overload. This study found that ETV1‐CKO was a key regulator of RyR2‐mediated calcium leakage in the sarcoplasmic reticulum, and that ETV1‐deficiency increased RyR2‐mediated CaTs and calcium leakage, prompting the occurrence of calcium overload.

### 4.4 Abnormalities of the ETV1/RyR2/CaMKII regulatory axis and reduction of *I*_Ca_ _,L_ drive AF formation

Down regulation of Cav1.2, shortened AP duration, increased frequency of afterdepolarizations and AF inducibility were found in ETV1 knockout mice. In addition, the enhanced RyR2 function in ETV1 knockout mice increased the intracellular calcium ions released from sarcoplasmic reticulum into the cytoplasm. CaMKII‐dependent phosphorylation of RyR2 contribute to AF. The underlying mechanism by which transcription factor ETV1 induced the expression of Cav1.2 and conversely elevated the expression of CaMKII and RyR2 remains unclear. In development, ETV1 performs physiological functions in branching morphogenesis, rapid conduction physiology in the heart and motor coordination.^45^ Accumulating data indicate a central role for the hepatocyte growth factor/tyrosine protein kinase Met (HGF/c‐Met) pathway in embryogenesis, cell growth and movement, tissue morphogenesis, morphological differentiation and organ regeneration. Upon binding to HGF, dimerization and trans‐autophosphorylation of c‐MET activate various downstream signalling pathways, including the ERK1/2/JNKs/p38 MAPK and the PI3K‐Akt pathway, which reach the nucleus to affect gene expression involving angiogenesis and metastasis.^46^ Metastasis‐related genes Met are downstream targets of ETV1. It has been suggested that HGF upregulated ETV1 via ERK1/2 pathway. HGF upregulated ETV1 expression via c‐MET‐ERK1/2‐ELK1 cascade, which in turn upregulated c‐MET expression, forming positive feedback.^47^ We deduce that the expression of Cav1.2, CaMKII and RyR2 is affected by ETV1 through c‐MET. AF is a heterogeneous disease and there can be variability in how the calcium handling proteins are expressed in different disease settings. The genetic background of an individual may be a critical determinant of how calcium handling moieties are disrupted to result in AF. As 178 potential ETV1 target genes have been identified and ETV1 shares binding sites with TBX5, NKX2.5 and GATA4 in atrial myocytes, future studies should focus on elucidating the interplay between ETV1 and other AF transcription factors to regulate calcium handling.^48^

## 5 LIMITATIONS

There are several limitations in this study. (1) Our research group did not perform dynamic electrocardiogram telemetry on conscious mice as conscious mice are more able to reflect normal physiological functions. (2) The APD shortening mainly stems from the shortening of the action potential plateau phase, which mainly consists of the inward L‐type calcium current (*I*_Ca,L_) and outward potassium currents (*I*_to_, *I*_kur_ and *I*_ss_) in the mouse atrium. Future studies need to focus on the outward potassium currents. (3) The ISO intervention should be added in the experiment to confirm PKA role in the RyR leak. (4) No clinical data were included, and the source of increased intracellular Ca^2+^ needs further studies. (5) Future studies need to focus on the pathophysiological features of AF associated with different aetiologies.

## 6 CONCLUSIONS

ETV1 deficiency has a significantly increased susceptibility to AF. The susceptibility to AF is associated with increased DADs, which originate from abnormal intracellular calcium metabolism. The APD shortening originated from decreased L‐type calcium current, which was closely associated with decreased Cav1.2 expression and increased intracellular calcium load in atrial myocytes of ETV1 deficiency mice. Elevated expression of the calcium transport‐related proteins RyR2, p‐RyR2 and CaMKII which is the molecular basis of the increased intracellular calcium load in ETV1 deficiency mice.

## AUTHOR CONTRIBUTIONS

**Li‐Hua Fang:** Investigation (equal); methodology (equal). **Qian Chen:** Writing – original draft (equal); writing – review and editing (equal). **Xian‐Lu Cheng:** Data curation (equal); formal analysis (equal); investigation (equal). **Xiao‐Qian Li:** Data curation (equal); formal analysis (equal). **Tian Zou:** Writing – original draft (equal); writing – review and editing (equal). **Jian‐Quan Chen:** Data curation (equal); investigation (equal); methodology (equal). **Guo‐Jian Xiang:** Investigation (equal); methodology (equal); software (equal). **Qiao Xue:** Formal analysis (equal). **Yang Li:** Supervision (equal). **Jian‐Cheng Zhang:** Conceptualization (equal); funding acquisition (equal); project administration (equal).

## FUNDING INFORMATION

This work was supported by grants obtained from the National Natural Science Foundation of China [82070341, 82370327], the National key research and development projects [2022YFA1104303‐2], the Natural Science Foundation of Fujian Province [2019J01189, 2020J011074, 2023J011189], Youth research projects of Fujian Provincial Health Commission [2020QNA006] and Starup Fund for scientific research of Fujian Medical University [2020QH1140].

## CONFLICT OF INTEREST STATEMENT

The authors declare no conflicts of interest.

## Data Availability

Data available on request from the authors.
